# ZrP_2_O_7_ as a Cathodic Material in Single-Chamber MFC for Bioenergy Production

**DOI:** 10.3390/nano12193330

**Published:** 2022-09-24

**Authors:** Abdellah Benzaouak, Noureddine Touach, Hanane Mahir, Youssra Elhamdouni, Najoua Labjar, Adnane El Hamidi, Mohammed El Mahi, El Mostapha Lotfi, Mohamed Kacimi, Leonarda Francesca Liotta

**Affiliations:** 1 Laboratory of Spectroscopy, Molecular Modeling, Materials, Nanomaterials, Water and Environment, Environmental Materials Team, École Nationale Supérieure d’Arts et Métiers (ENSAM), Mohammed V University in Rabat, Rabat 10 000, Morocco; 2 Laboratory of Physical Chemistry of Materials, Catalysis and Environment, Department of Chemistry, Faculty of Sciences, Mohammed V University in Rabat, Rabat 10 000, Morocco; 3 Istituto per lo Studio dei Materiali Nanostrutturati (ISMN)-CNR, via Ugo La Malfa, 153, 90146 Palermo, Italy

**Keywords:** pyrophosphate, MFC, wastewater treatment, energy production

## Abstract

The present work is the first investigation of the electrocatalytic performances of ZrP_2_O_7_ as a cathode in a single-chamber Microbial Fuel Cell (MFC) for the conversion of chemical energy from wastewater to bioelectricity. This catalyst was prepared by a coprecipitation method, then characterized by X-ray diffraction analysis (XRD), Fourier transform infrared spectroscopy (FTIR), scanning electron microscopy (SEM), energy dispersive X-ray analysis (EDX), ultraviolet–visible–near-infrared spectrophotometry (UV–Vis–NIR), and cyclic voltammetry analyses. The acid–basic characteristics of the surface were probed by using 2-butanol decomposition. The conversion of 2-butanol occurs essentially through the dehydrating reaction, indicating the predominantly acidic character of the solid. The electrochemical test shows that the studied cathode material is electroactive. In addition, the ZrP_2_O_7_ in the MFC configuration exhibited high performance in terms of bioelectricity generation, giving a maximum output power density of around 449 mW m^−2^; moreover, it was active for wastewater treatment, reducing the chemical oxygen demand (COD) charge to 50% after three days of reaction.

## 1. Introduction

ZrP_2_O_7_ is a material of interest due to its optical, optoelectronic, and catalytic properties [1,2,3,4,5,6,7]. It is an isotype compound of tetravalent metal pyrophosphates MP_2_O_7_ (M = Ti, Zr, Hf, Ge) [8]. This material belongs to a class of inorganic materials that is of great interest given their application potential, in particular, in the fields of battery development [9]. The highest symmetry possible for MP_2_O_7_ structures is Pa-3, due to the symmetry of the pyrophosphate group [10]. This solid has important thermal properties, particularly its low coefficient of thermal expansion at high temperatures due to its low thermal conductivity, making it an ideal light insulator [11]. It should be noted that the compound ZrP_2_O_7_ has a typical anisotropic chemical bonding character [12], which can make it a candidate as a catalyst or catalyst support [4,13,14]. Further, its properties as an electrode for batteries and fuel cells have been studied [9,15], from which our idea of studying the feasibility of this solid in microbial fuel cells (MFCs) arose. Indeed, G. Hu et al. [9] reported that a ZrP_2_O_7_ electrode coating plays a critical role in battery performance. It could contribute to the removal of residual lithium from electrode surfaces, prevention of electrolyte erosion, and suppression of interfacial side reactions, as it can also increase the diffusion coefficient of lithium ions via reducing the electrode polarization.

Recently, MFCs have gained a large amount of attention in the literature because they represent a new approach for reducing the amount of wastewater and producing electricity simultaneously [16]. The principle of this bio-electrochemical system is to convert biodegradable organic matter into electricity by the action of microorganisms. MFCs produce electrons and protons at the anode, then the protons pass through a proton exchange membrane (PEM) to the cathode to combine with electrons collected by an external circuit and an electron acceptor, usually oxygen, where the main oxygen reduction reaction (ORR) occurs.

In order to have an efficient and cost-effective implementation of MFC devices at a high scale, some issues must be addressed, such as power output when increasing wastewater volume, the feedstock characteristics [17], the design and cost of the electrodes [18]. The electrodes (anode and cathode) and the membrane are the main components of this device. The most studied anodes and membranes are graphite and Nafion, respectively [19]. Regarding the cathode, which is the catalyst for the oxygen reduction reaction, the most well-known is platinum (Pt); however, this material suffers from poisoning problems and high cost [20]. Since the performance and cost of MFCs are directly related to the performance of these materials [21], for this purpose, many investigations have been carried out to design new nonprecious cathode materials for MFCs [22]. In addition, as mentioned above, ZrP_2_O_7_ has attracted great interest due to its performance as an additive of electrodes in batteries and in DMFC membranes [9,23].

It should be noted that the catalytic behavior of different varieties of zirconium phosphate has been widely studied, for both amorphous and crystalline phases, in particular α-ZrP, which is a strong acidic catalyst [24]. The dehydration of alcohols and the adsorption of probe molecules are adequate for characterizing the acid–base character [25,26]. It has been reported that the acidity of this type of material is related to the polar P–OH bonds on the surface and the highly electronegative oxygen atoms located around the phosphorus atoms.

The main purpose of the present work is to introduce a new type of cathode, based on ZrP_2_O_7,_ for microbial fuel cells to convert chemical energy from wastewater into electricity. This is a preliminary study of the electrocatalytic performances of ZrP_2_O_7_; however, considering the low cost and availability of phosphates in Morocco, such materials may favor the implementation of MFCs in the production of bioenergy at a large scale.

## 2. Materials and Methods

### 2.1. Catalyst Preparation

For the preparation of ZrP_2_O_7_ material, the stoichiometric amount of ZrOCl_2_·8H_2_O was added to 50 mL of distilled water and stirred to completely dissolve. Then, (NH_4_)_2_HPO_4_ previously dissolved in 50 mL of distilled water, following a ratio of P/Zr = 2, was added dropwise. The solution was heated at 90 °C; then, a few drops of ammonia solution were added to adjust the initial pH value (~1) to 2. The temperature was kept constant at 90 °C for one hour. The mixture was dried at 120 °C in an oven for 24 h and the resulting solid was calcined at 700 °C for 4 h.

### 2.2. Characterization

X-ray diffraction (XRD) was used to investigate the crystalline structure of the ZrP_2_O_7_ compound. XRD analysis was performed on a Bruker D8 Advance diffractometer (Germany) with Cu-Kα radiation in the range 10–80° 2θ, by using a 0.02° step size and a scan speed 0.05°·s^−1^. The crystalline phases were attributed by using the ICDD database.

The optical properties were investigated by UV–visible–NIR diffuse reflectance spectra at room temperature within the range of 190–1000 nm using a spectrometer type Varian Cary 5-E (Australia) with an integrating sphere coated with polytetrafluoroethylene (PTFE) and double monochromator. PTFE was also used as a reference.

Fourier transform infrared spectra (FTIR) were recorded in the wave-number range between 4000 and 400 cm^−1^ using a VERTEX-70 spectrophotometer with 4 cm^−1^ resolutions.

Analyses of morphology, composition, and elemental mapping distribution were performed using scanning electron microscopy (SEM) and energy dispersive X-ray (EDX) analysis on QUATTRO S-FEG-Thermo Fisher equipment.

The electrochemical tests were carried out by using a BioLogic Scientific Instruments SP-150 potentiostat equipped with EC-Lab software and a three-electrode system.

2-Butanol conversion was used as a probe reaction to evaluate the acid–base properties of the catalyst. The study was carried out between 120 and 240 °C in a U-shaped continuous-flow microreactor operating at atmospheric pressure. 2-Butanol diluted in N_2_ flow was fed into the reactor at a partial pressure of Pol = 8.4 × 10^2^ Pa, fixed using a saturator whose temperature was controlled by a thermostat. The total flow rate was 60 cm^3^ min^−1^. To avoid any condensation of the reagent, the stainless-steel pipes were heated to 60 °C. The reaction mixture was analyzed by an FID chromatograph equipped with a stainless-steel column (diameter 1/8 inch) containing Carbowax 1500 (15%) on Chromosorb PAW (60/80 mesh). The yield was calculated as follows:

(1) $$\mathrm{yield}\;\%\;=100\;\times \frac{\mathrm{moles}\;\mathrm{of}\;\mathrm{product}\;i}{\mathrm{initial}\;\mathrm{number}\;\mathrm{of}\;\mathrm{moles}}$$

### 2.3. MFC Configuration

The performance of ZrP_2_O_7_ was evaluated in a single-chamber MFC, consisting of a double-jacketed glass reactor to ensure thermal stability with an anodic capacity of 250 mL. The cathode consisted of ZrP_2_O_7_ oxide mixed with a 60 wt% of polytetrafluoroethylene (PTFE) as a binder (Sigma-Aldrich, Darmstadt, Germany) dispersed in water and isopropanol. The mixture was mechanically pressed against a carbon cloth surface of 4 cm diameter, without exceeding a surface of 1 cm^2^. The mass ratio of PTFE:catalyst was 1:9, for a total loading of 60 mg·cm^−2^. The anode consisted of 100 g of graphite particles with a diameter of 2–6 mm and a graphite rod (Graphite Store) with a diameter of 3.18 mm, which was connected to the cathode with a resistance of 1 kΩ. All of the tests of MFC were carried out in a batch reactor. The anode chamber was fed with a mixture of domestic waste and sewage sludge (125 mL) at a COD load of 3483 mg L^−1^ and a pH of 7.88.

For the separation, the Nafion^®^ polymer, as a proton exchange membrane (PEM) of 4 cm diameter, was positioned between the cathode and the anode compartment [27].

The polarization curves and power output profiles were constructed by varying the external resistance from 11 MΩ to 1 Ω. The power (P) and current (I) densities were estimated by using the equations I = V/R and P = V^2^/R, where V is cell voltage and R is an external resistor, and then normalized to the geometric cathode area. Internal resistance was calculated from the power curve at the maximum power point. The effectiveness of wastewater treatment was evaluated by chemical oxygen demand (COD) abatement. COD was measured according to the APHA protocol [27,28] using a Spectroquant Nova 30 spectrophotometer.

## 3. Results and Discussion

### 3.1. Materials Characterization

#### 3.1.1. XRD Analysis

**Figure 1:**
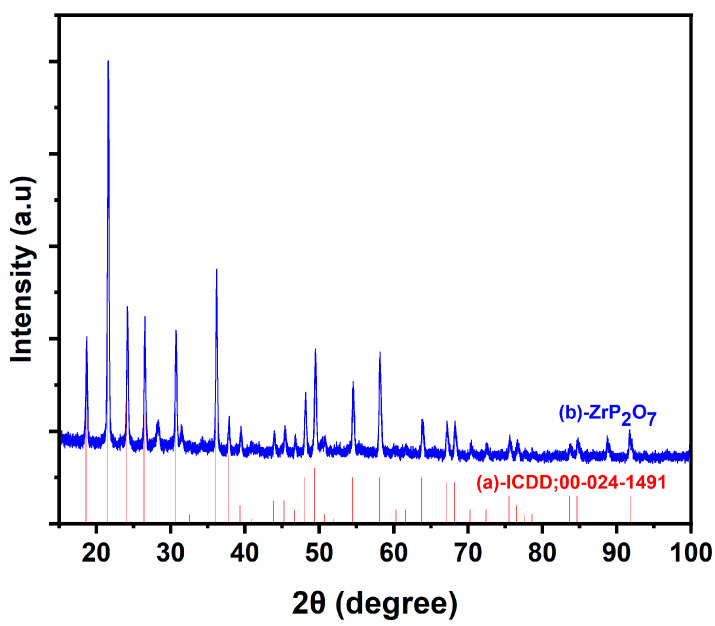
XRD pattern of ZrP_2_O_7_.

Figure 1 depicts the X-ray diffraction pattern of the ZrP_2_O_7_ material. The results show that the obtained product is composed of a single phase matching the JCPDS Card No: 24-1491. The XRD peaks were indexed in the cubic system having parameter a = 8.241 A°.

#### 3.1.2. UV–Visible–Near-IR Spectroscopy

**Figure 2:**
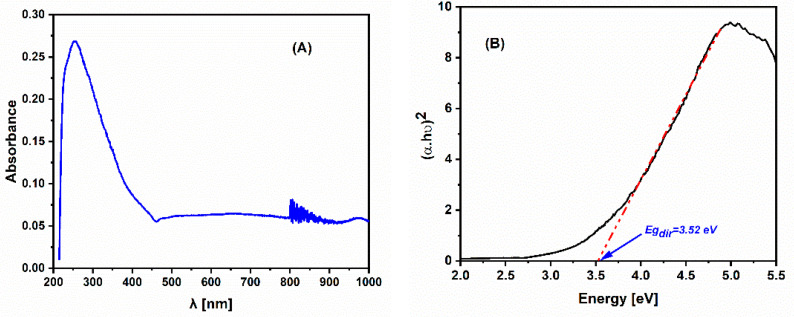
UV–Visible–near-IR absorbance of ZrP_2_O_7_ (**A**) and Tauc’s plot (**B**).

The optical properties of ZrP_2_O_7_ were investigated by recording the absorbance spectrum of this solid in the UV–visible–near-infrared range. The optical energy gap was determined at room temperature from Tauc’s plot [29], using the following equation:

(2) $$\alpha h\nu =A{\left(h\nu -E_{g}\right)}^{n}$$

where α represents the absorption coefficient, hν is the incident photon energy, A is a constant of proportionality, Eg denotes the band gap energy, and n is the power factor of the transition mod; it is equal to ½ for the direct allowed transition and 2 for indirect allowed transition. Additionally, the intersection with the x axis of the straight-line part of the plot of ${\left(\alpha h\nu \right)}^{2}$ (red dashed line, Figure 2B) as a function of the incident energy provides the value of the direct band gap.

Figure 2A displays the UV–vis–near-IR absorption spectrum of ZrP_2_O_7_. It presents a characteristic peak of absorbance in the UV range with a maximum located at around 255 nm, which can be attributed to the O^2−^ → Zr^4+^ charge-transfer band. The direct band gap energy of this material, determined from Tauc’s plot in Figure 2B, is equal to 3.52 eV at room temperature. The utility of band gap energy as a descriptor for catalyst oxidation activity [30], as well as for oxygen reduction and evolution reactions on perovskite oxides, has been demonstrated as a key parameter for the design of materials with good adsorption properties of oxygen species [31]. With this aim, we have determined the band gap energy at room temperature for ZrP_2_O_7_ to report an important parameter for identifying the electronic properties of such a material that is useful for understanding the electrochemical activity.

#### 3.1.3. FTIR Analysis

**Figure 3:**
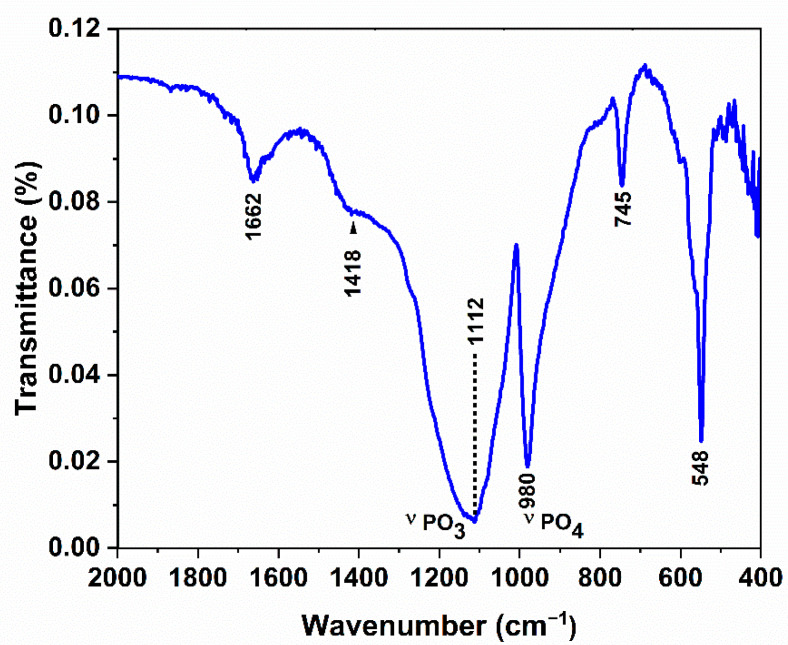
FTIR spectrum of ZrP_2_O_7_.

The FTIR spectrum corresponding to the ZrP_2_O_7_ compound is shown in Figure 3. Large broad bands occur at 3434 cm^−1^ associated with a weak peak around 1662 cm^−1^ and are attributed to the water O–H stretch [32]. The shoulder band of moderate intensity at 1418 cm^−1^ is ascribed to δ(POH) [33]. The P_2_O_7_^4−^ group frequencies are assigned according to the PO_3_ groups and P–O–P bridge vibrations. According to Guler and al. [34], the frequency of P–O in PO_3_ vibration is expected to be higher than that of the P–O–P bridge, since the P–O bond of the PO_3_ group is stronger than the P–O–P bridge. The broad and intense band centered around 1112 cm^−1^ includes both symmetric and antisymmetric vibrations of the P–O bond of PO_3_. The observed band at 980 cm^−1^ can be assigned to the symmetrical stretching vibrations of PO_4_ [35]. The 745 cm^−1^ vibrations are assigned to POP stretching [32]. The intense band observed at 548 cm^−1^ located in the 595–464 cm^−1^ frequency region is attributed to the deformation bands of δ(OPO), δ(PO_3_), and δ(POP) [34].

**Figure 4:**
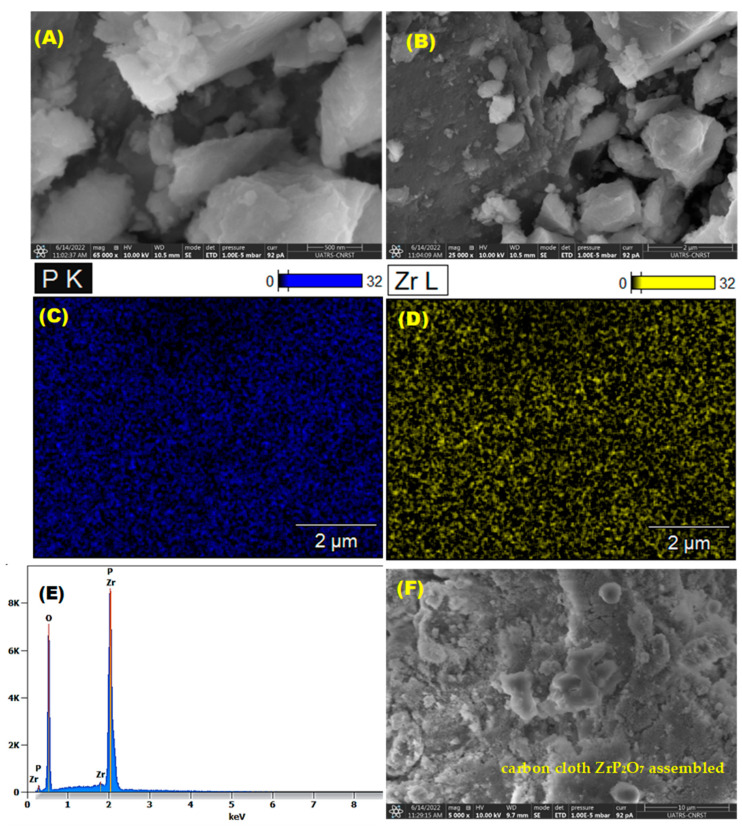
SEM Micrographs (**A**,**B**), EDX spectral (**E**) and elemental distribution analysis (**C**,**D**) of ZrP_2_O_7_ and carbon cloth-assembled ZrP_2_O_7_ (**F**).

The microstructures of the active phase and carbon cloth-assembled ZrP_2_O_7_, analyzed by SEM, are shown in Figure 4. The ZrP_2_O_7_ phase is composed mainly of grains of different sizes. The mapping from EDX analysis illustrates a homogeneous dispersion of Zr and P. Spectral and semiquantitative analyses of the composition are given in Figure 4 and Table 1, respectively.

### 3.2. Catalytic Properties and Electrochemical Performances of ZrP_2_O_7_

#### 3.2.1. Decomposition of 2-Butanol

The acid–base character of divided metal oxides is generally involved in explaining their catalytic properties [36]. Catalytic activity and catalyst selectivity in specific reactions are widely used to characterize the acid–base and redox properties of catalysts. The decomposition of 2-butanol has been used here as a probe of the acid–base properties of the studied solid surface. Dehydration and dehydrogenation of alcohols are often used for the determination of acidity and basicity of catalysts, respectively. Dehydration is usually strictly related to the acidity of the catalysts, while, concerning the basicity evaluation, dehydrogenation does not seem to give unequivocal results since it has been previously reported that this reaction requires redox sites [37,38].

**Figure 5:**
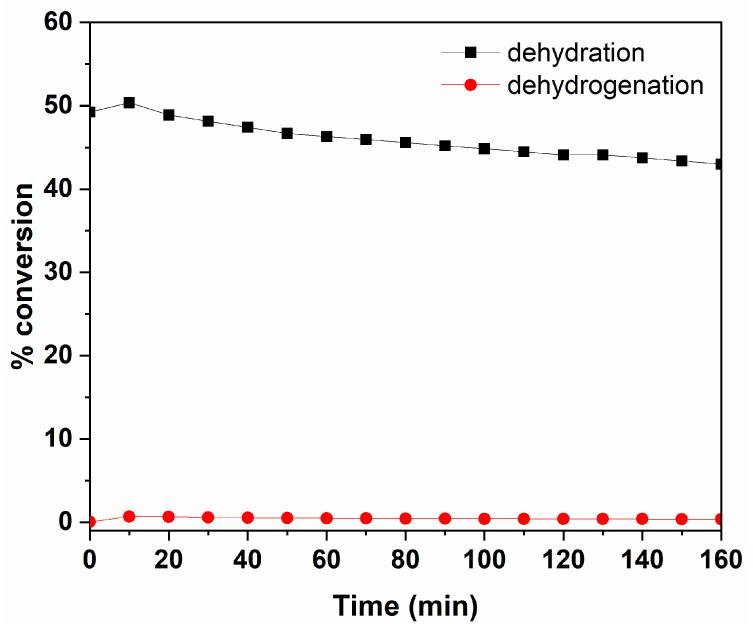
Dehydrating and dehydrogenating curves of 2-butanol over ZrP_2_O_7_ under N_2_ at 185 °C (in absence of oxygen) as a function of time.

When operating with a reaction mixture of 2-butanol and N_2_ at 185 °C, butenes are practically the only products of the reaction. Figure 5 illustrates the 2-butanol conversion curves describing the extent of dehydrating and dehydrogenating reactions over ZrP_2_O_7_ as function of the time. It has been found that the dehydrating of 2-butanol leading to butenes is the most important reaction, with a conversion close to 50%, slightly decreasing to ~45% during the reaction. On the other hand, the low formation of butanone via dehydrogenating activity is due to the acidic nature of the surface, either a Bronsted or a Lewis acidity type.

Previous studies have been carried out using alcohol dehydration (isopropanol, 1- or 2-butanol) and butene isomerization to investigate the acidity and the derived phases of zirconium phosphate prepared by different methods or by ion exchange with Na^+^, Cs^+^, or Ag^+^ [39,40,41]. It is reported that the active sites of both ZrP_2_O_7_ and Zr(HPO_4_)_2_.x H_2_O phases have a predominance of Bronsted sites on the surface, demonstrated by a strong decrease in their catalytic activity after surface poisoning by Cs^+^. Interpreting the results of these studies, the acidic sites of such catalysts generally have a medium strength.

#### 3.2.2. Electrocatalytic Activity in Single-Chamber MFC

**Figure 6:**
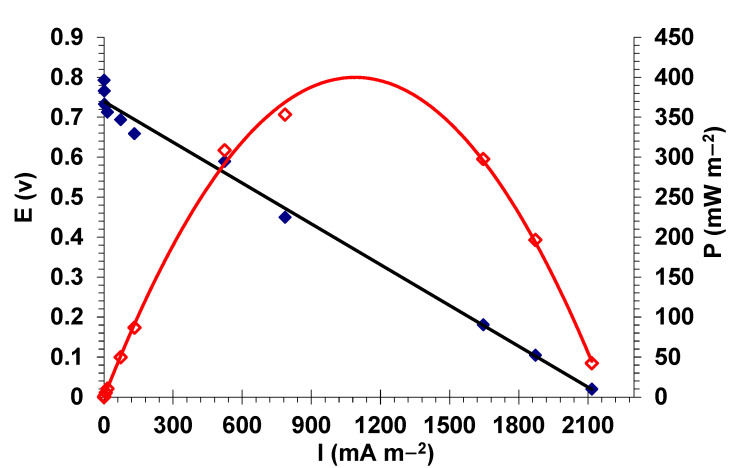
Power density and Polarization curves of MFCs with ZrP_2_O_7_ catalyst.

The voltage versus current density relationship in microbial fuel cells is generally described by polarization curves [42]. For the studied system, the polarization curve and power output profile for the closed-circuit air-cathode MFC based on the ZrP_2_O_7_ catalyst are shown in Figure 6. The highest open-circuit voltage recorded for this MFC device was 793 mV, obtained during the first three day’s functioning. As illustrated in Figure 6, the MFC is a voltage generator with an almost-linear characteristic curve V(I). Generally, this characteristic curve presents two points of inflection, dividing the curve into three parts: low currents corresponding to the voltage drop of activation, the voltage drop due to the concentration limit phenomena at high currents, and ohmic loss in the electrolyte corresponding to the linear part of the curve [43,44]. The drop in the voltage with the increasing current density is due to the ohmic resistance related to the wire connections and materials. Indeed, the ohmic losses in a MFC include the resistance to electron flow through the electrodes, the interconnections, and the anode electrolyte [42,45]. Since the voltage drop of the MFC operating with ZrP_2_O_7_ is relatively linear with current, ohmic losses would dominate in this zone [43]. These results are in good agreement with those observed in power curves. The ZrP_2_O_7_ catalyst used in an air-cathode MFC achieves a power density of 449 mW·m^−2^ with an associated current density of 1220 mA·m^−2^, which is a very encouraging result when compared with recently studied MnO_2_-based cathodes recognized as potential cathode materials for microbial fuel cells [46,47,48]. In other recent studies, Aicha Zerrouki et al. [49] synthesized organometallic (Raney nickel and Fe-complex)-based cathodes, achieving a maximum power output around 39 mW·m^−2^, significantly lower than that of the ZrP_2_O_7_-based cathode studied in this work. On the other hand, Liting Jiang et al. [50] have tested other types of nickel–iron-based double hydroxide (NiFe-LDH)-based cathodes. The authors reported that air-cathode MFCs using cathodes coated with core–shell-structure cobalt trioxide combined with NiFe-LDH (Fe_3_O_4_@NiFe-LDH) reached a maximum power density of 211.4 mW·m^−2^ [50]. Shengnan Li et al. [51] employed a class of MOF materials containing metallic nickel as the air-cathode MFC and its performance, in terms of the power output of 446 mW/m^−2^, is higher than that obtained by Liting Jiang et al. (211 mW·m^−2^). The performance of ZrP_2_O_7_ as a catalyst in single-chamber air-cathode MFCs is better than that obtained for these types of alternative materials [50,51,52,53].

**Figure 7:**
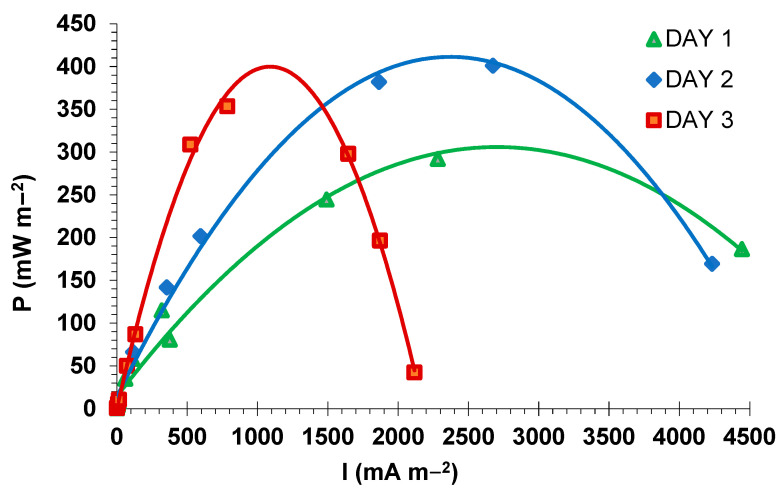
Power density curves depending on current density per day during the MFC functioning with the ZrP_2_O_7_ catalyst.

The maximum power output, for three days of the experiment, was determined by the polarization curves, as presented in Figure 7. The power output recorded on the first, second, and third day was 308, 418, and 449, respectively.

Regarding the treatment of effluents, the chemical oxygen demand (COD) was measured before and after the experiment. The abatement of the COD, measured after three days of the experiment, shows that the initial value (3483 mg/L) was reduced by 50.5% (1721 mg/L).

As can be observed from Figure 7, the results display that the power density continues to increase progressively depending on the time, due to the very high organic charge of the treated wastewater, which has not been totally reduced during the experiment. On the other hand, it was found, according to Figure 7, that the limiting current of the cell decreases as a function of time accompanied by an increase in the internal resistance (Table 2) due to the loss in concentration [54].

The so-far-discussed performances of ZrP_2_O_7_ as a cathodic material were attributed to a synergy of zirconium phosphate with Nafian, facilitating proton conduction [23]. Indeed, the presence on the catalyst surface of OH groups with some Bronsted-acidic character, as has been demonstrated by 2-butanol dehydration and the acid–base interaction between the sulfonic acid groups of Nafion with the O_3_POH moieties, is well-known in the literature [55]. The Lewis-acidic character of Zr sites in ZrP_2_O_7_ [56] able to activate O_2_ may further enhance the electrochemical activity, favoring the oxygen reduction reaction (ORR) at the cathode. Moreover, the band gap of 3.52 eV, lower than the 6.4 eV previously reported for ZrP_2_O_7_ by other authors [57], correlates well with the good electrochemical performances of ZrP_2_O_7_ as a cathode in MFCs.

#### 3.2.3. Electrochemical Characterization

**Figure 8:**
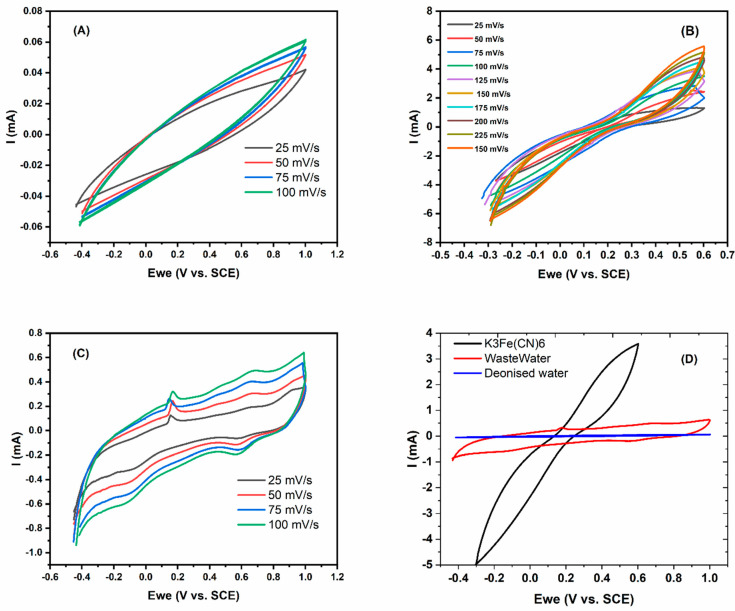
Cyclic voltammograms of ZrP_2_O_7_ catalyst with various scan rates in (**A**) denoised water, (**B**) wastewater, (**C**) ferrocyanide solution, and (**D**) comparison of the three experiments at 100 mV/s.

The electrochemical properties of ZrP_2_O_7_ material were examined by cyclic voltammetry (CV) in three different electrolytes (distilled water, wastewater, and K_3_Fe(CN)_6_) using a three-electrode cell. Figure 8A–D shows the voltammograms of the studied catalyst, at different scan speeds, in the three conditions. It has been noted that as the scan speed increases, the peaks became more appreciable and larger. This was observed in increasing order from deionized water through wastewater to iron cyanide, with clear redox peaks for the latter. This tendency was differentiated when the three CV tests were performed at a sampling rate of 100 mVs^−1^ in a potential range of −0.3–1 V (vs. SCE). Additionally, the ZrP_2_O_7_-based electrode showed larger electroactive areas in wastewater and ferrocyanide solutions compared to denoised water, which may allow practical applications for wastewater purification and energy production in MFC devices. Indeed, the shape of the voltammograms in this study is in agreement with those reported in the literature when the wastewater was degraded using similar graphite electrodes in MFC devices. In contrast, the peaks in the voltammograms were not clearly observed [58,59,60].

## 4. Conclusions

This work is a preliminary investigation on the use, for the first time, of ZrP_2_O_7_ in microbial fuel cells. Summarizing the results, we can draw the following conclusions:

ZrP_2_O_7_ has been assessed as a cathode in a single-chamber MFC for wastewater treatment and electricity generation.

The electroactivity properties of ZrP_2_O_7_ were ascribed to a good synergy between OH groups of zirconium phosphate with Nafion, facilitating proton conduction.

The presence of Zr Lewis-acidic sites, able to activate oxygen on the cathode surface, may favor the oxygen reduction reaction, further improving the electrochemical performances.

The band gap energy value was calculated as a descriptor for the cathodic electrochemical activity of ZrP_2_O_7_.

High power density and 50% OCD reduction in the treated wastewater were found.

The ZrP_2_O_7_ oxide compares well with alternative cathode materials reported in the literature for single-chamber MFC applications.

## Figures and Tables

**Table 1:** Composition by EDX analysis.

	Weight %	Atom %
**O K (S)**	42.8	70.1
**P K**	24.2	20.5
**Zr L**	33.0	9.5

**Table 2:** Max power and internal resistance of the studied system per day.

Day	P_max_ (mW/m^2^)	R_in_ (ohm)
**1st**	308.6	416.74
**2nd**	418.06	720.64
**3th**	448.92	2998.92

## Data Availability

Not applicable.
